# A Case Report on Juvenile Folliculotropic Mycosis Fungoides: A Misleading Disease

**DOI:** 10.7759/cureus.82009

**Published:** 2025-04-10

**Authors:** Lamia Mansour Billah, Awatef Kelati

**Affiliations:** 1 Dermatology Unit, Cheikh Khalifa International University Hospital, Casablanca, MAR

**Keywords:** atopic dermatitis (ad), case report, dermoscopy, folliculotropism, mycosis fungoides (mf)

## Abstract

Mycosis fungoides (MF) is the most common form of cutaneous T-cell lymphoma, usually affecting elderly patients. Folliculotropic mycosis fungoides (FMF) is a subtype of MF that affects the hair follicles. While it is the most common variant in adults, it is rarely described in the pediatric population. The clinical manifestations of FMF can mimic common childhood dermatoses, like atopic dermatitis (AD), which often leads to a delay in diagnosis. In the pediatric population, FMF is usually diagnosed at an early stage, and its course is often indolent. There are no guidelines for pediatric FMF; treatment is generally based on the approach used in adults and responds well to phototherapy combining narrow band ultraviolet B (NbUVB) and UVA. We report the case of an 11-year-old child who was treated for several years for atopic dermatitis with follicular involvement, revealing an FMF. This case highlights the value of clinicians' persistence in diagnosing and managing such a rare condition, as well as the essential role of the dermoscopic tool in identifying patterns that may improve the early diagnosis of MF.

## Introduction

Mycosis fungoides (MF) is the most common form of cutaneous T-cell lymphoma, typically affecting elderly patients. Folliculotropic mycosis fungoides (FMF) is a subtype of MF that involves the hair follicles. While FMF is the most common variant in adults, it is rarely reported in the pediatric population. The clinical manifestations of FMF can mimic common childhood dermatoses, such as atopic dermatitis (AD), which often leads to a delay in diagnosis [1,2]. We here describe an 11-year-old child who had been treated for several years for AD with follicular involvement and was ultimately diagnosed with FMF. This case highlights the challenges in the early diagnosis and management of this rare condition, highlighting the importance of clinicians' vigilance in diagnosing and managing FMF.

## Case presentation

**Figure 1:**
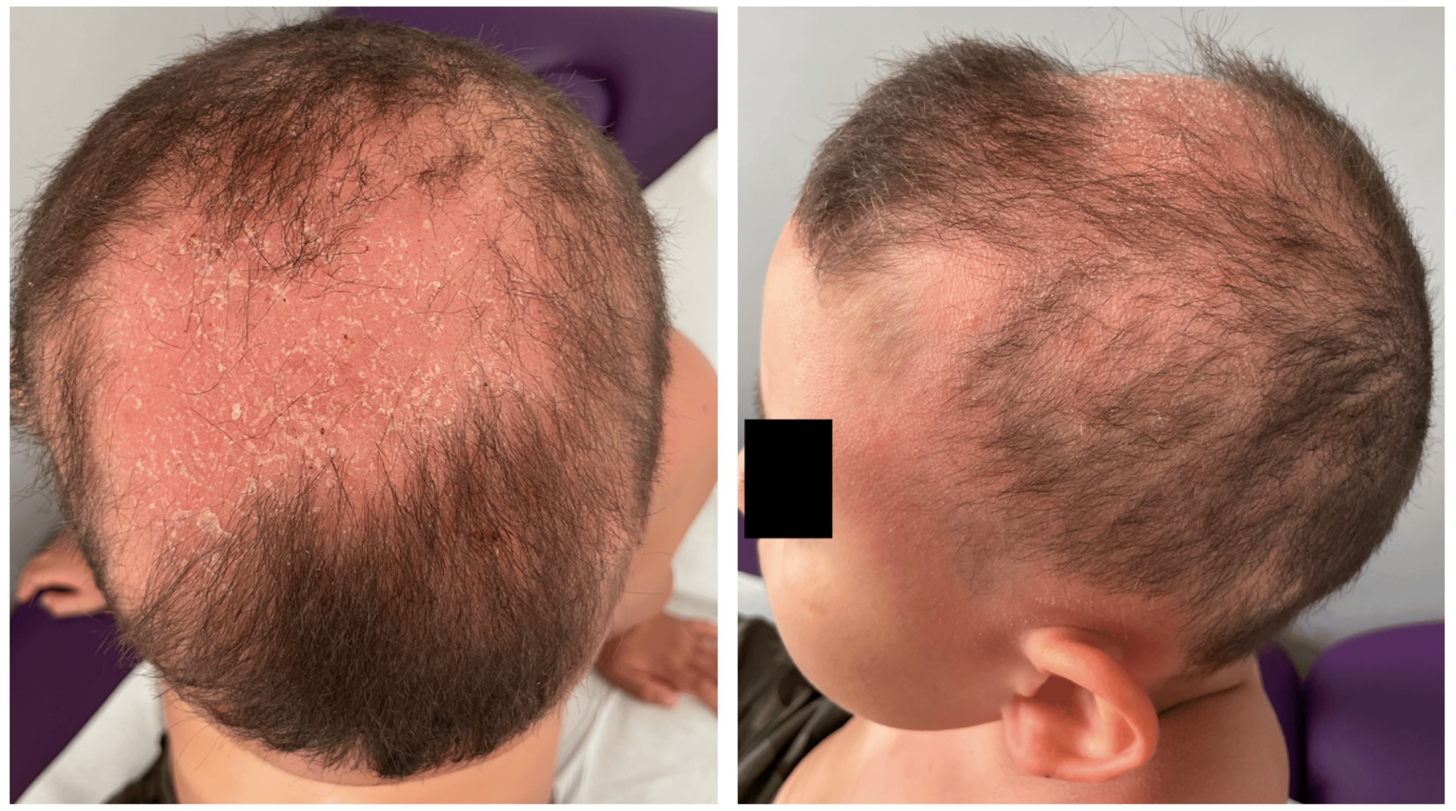
Alopecic and erythematosquamous scalp plaque

We report the case of an 11-year-old boy with a history of AD since childhood. He had a personal and family history of atopy, including allergic rhinitis. The patient presented with multiple pruritic skin plaques on his forearms, neck, and back, which had been treated with topical steroids for the past four years. The course was made of flare-ups and remissions. The flare-ups showed no seasonal patterns, and no aggravating factors were identified. Despite being treated with high-potency topical steroids, his symptoms persisted and worsened, with the appearance of an alopecic plaque on his scalp. On initial visit, clinical examination revealed a decrease in hair density with an alopecic, non-infiltrated, erythematous, and squamous plaque involving 45% of the scalp area (Figure 1).

**Figure 2:**
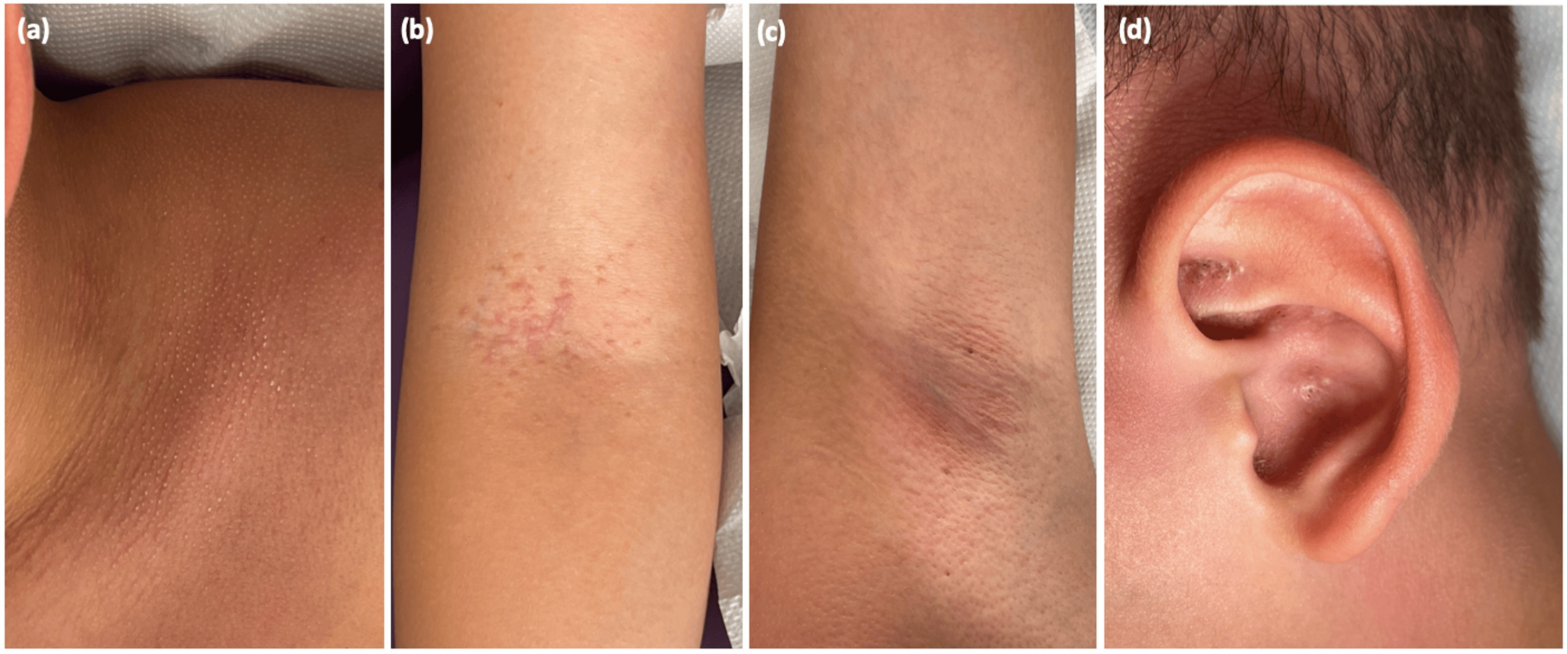
Follicular keratosis on an erythematous base on the trunk (a), armpits (b), axilla (c), with multiple comedo-like plugs on the ears (d)

**Figure 3:**
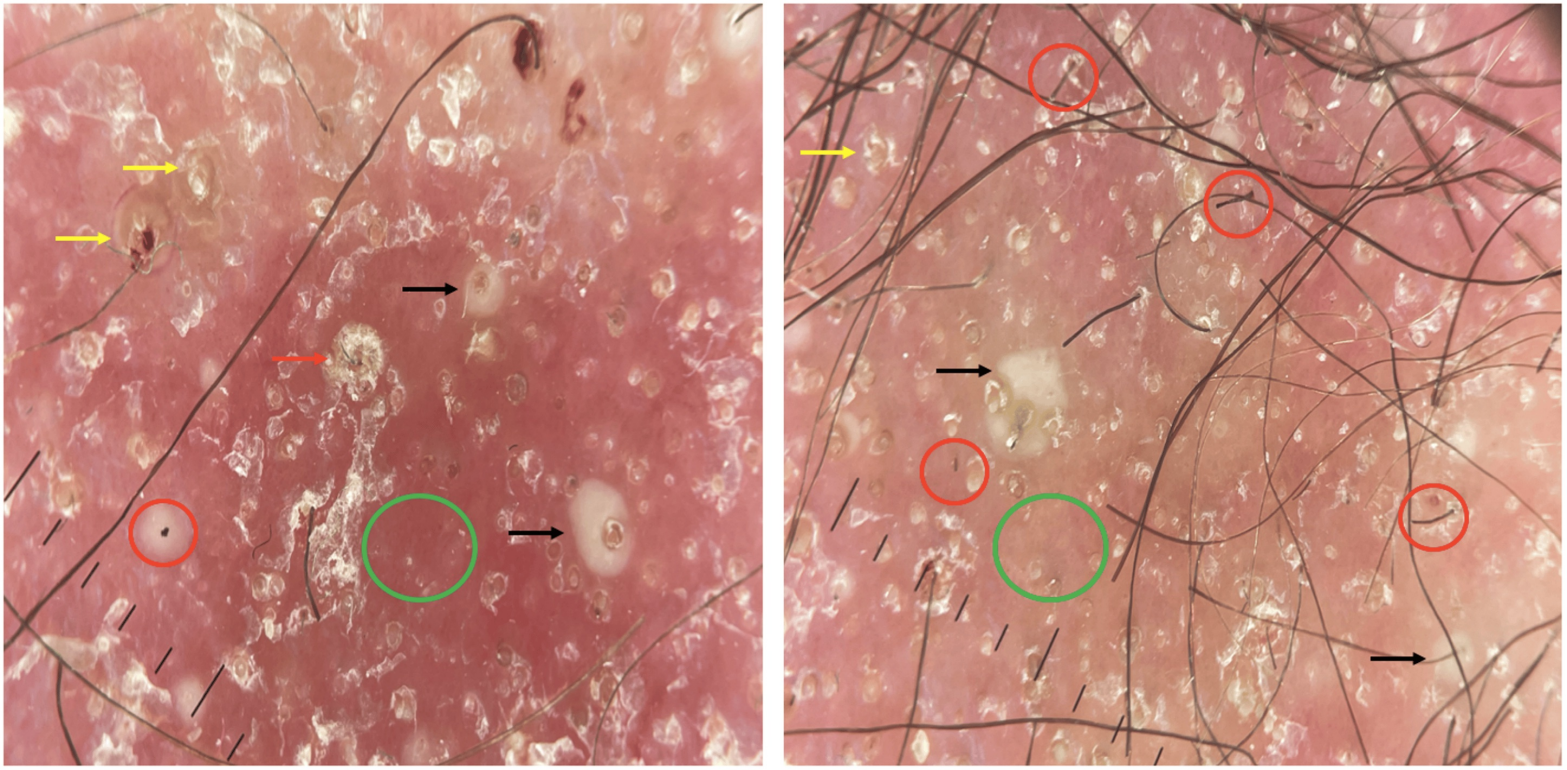
Dermoscopic images of the alopecic scalp plaque Follicular plugs (yellow arrow), peripilar scales (red arrow), with broken hairs (red circle), follicular pustules (black arrow), structureless white-yellowish areas replacing lost hair follicles (green circle), on an erythematous background

We also noted body depilation with follicular keratosis on an erythematous base on his face, trunk, back, neck, and armpits. Multiple comedo-like plugs were found on his ears (Figure 2). He had no palpable peripheral lymphadenopathy. Dermoscopy features of the scalp revealed follicular plugs, peripilar scales with broken hairs, follicular pustules, structureless white-yellowish areas replacing lost hair follicles, short and dotted vessels on an erythematous background (Figure 3).

**Figure 4:**
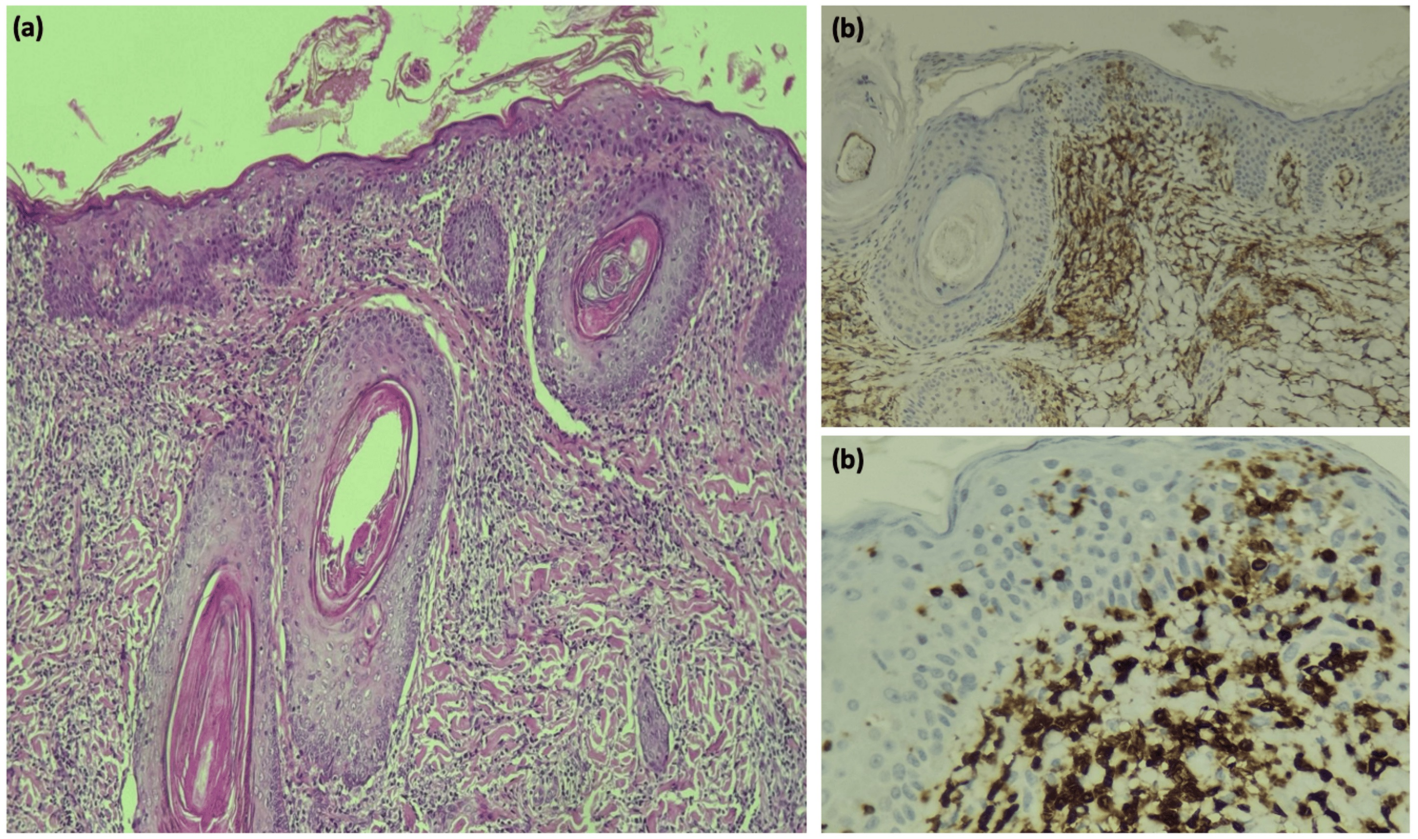
Histopathology features H & E: hematoxylin and eosin H & E stained at 10x magnification exhibiting a subepidermal perifollicular, periannexal lymphocytic infiltrate and epidermotropism (a). Immunohistochemistry for the T cell markers CD3, CD4, and CD8 (b)

Blood tests (blood count, peripheral blood smear, renal and liver function tests, IgE levels, serologies, and serum protein electrophoresis) were all normal. Chest X-ray and CT scan revealed no signs of systemic involvement. A core needle biopsy of the right axillary lymph node did not reveal any tumor cells. Multiple skin biopsy samples were taken from the alopecic plaque on the scalp and the axillary lesions. Histopathologic and immunophenotype features were consistent withFMF (Figure 4). The patient was classified as Stage IIa (T2N0M0). 

**Figure 5:**
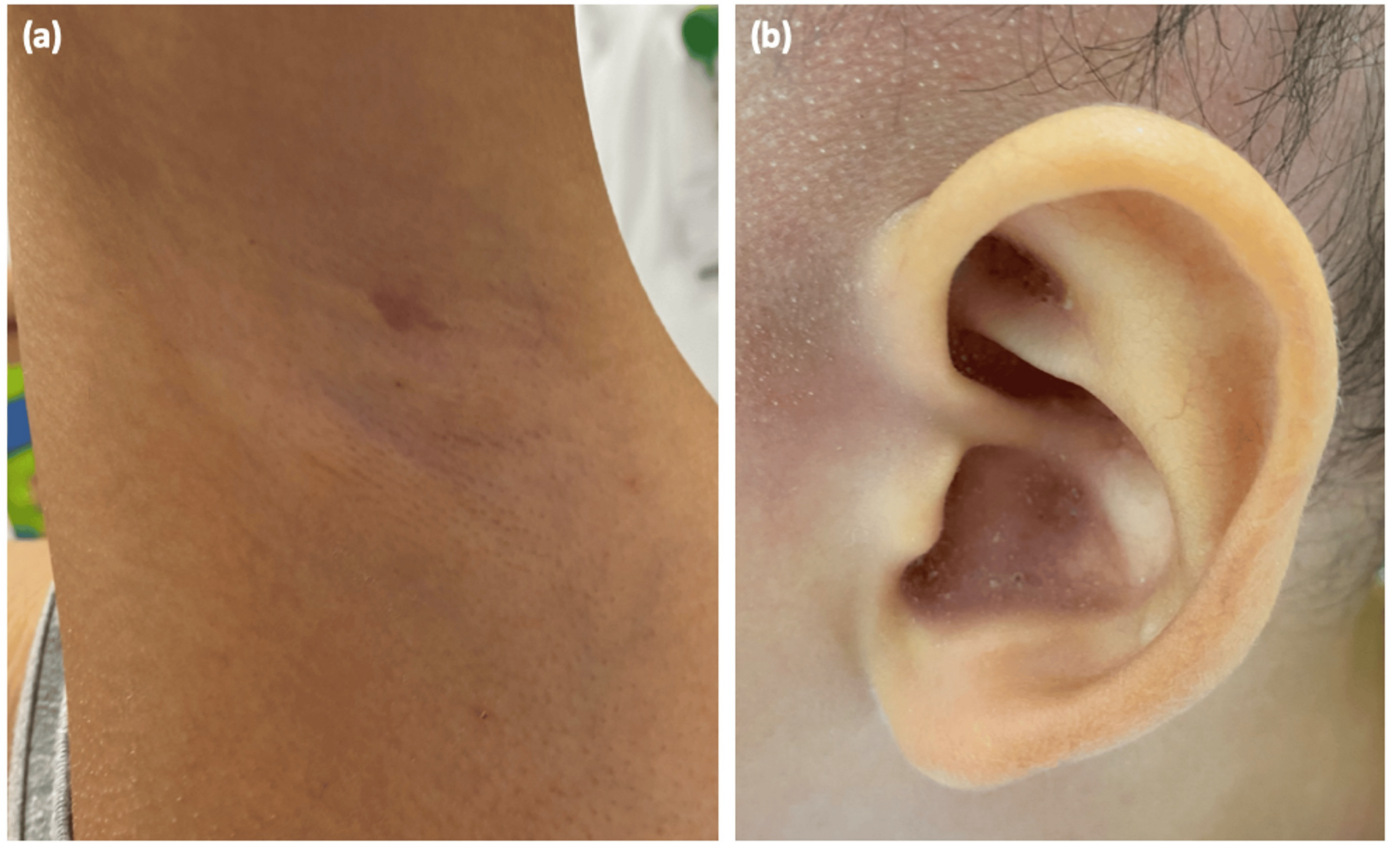
Clinical evolution Regression of follicular papular lesions in the axillary region (a) and the resolution of comedonal lesions on the ear (b)

He was treated with topical steroids and methotrexate injections at a dose of 15 mg per week. Four months later, he was seen with marked improvement in all the lesions, and he had had no new lesions since the treatment initiation (Figure 5).

## Discussion

The coexistence of both AD and MF in this case highlights the complex nature of dermatological diagnoses. AD is a chronic skin disease with recurrent inflammatory and pruritic lesions. Its pathogenesis involves genetics, immune responses, and environmental factors [3]. AD is often associated with a history of atopy, and its remission during adolescence is frequent but does not exclude the possibility of MF occurring [4]. Our case shows the importance of considering MF as a possible diagnosis, even in people with an atopic history [3].

The literature on juvenile MF is limited. Clinical features of MF in children are comparable to those observed in adults [1]. However, there are variations in the prevalence of disease types: hypopigmented MF is the most common form in the pediatric population, while the folliculotropic variant, which is common in adults, has rarely been described in children [4].

FMF is a variant of MF caused by the infiltration of atypical T lymphocytes into the hair follicles. Clinically, it manifests as follicular lesions mainly affecting the head and neck [5]. In its early stages, FMF presents as localized or diffuse follicular papules forming non-infiltrated plaques. A spiky or acne-like appearance has also been described [5]. In the advanced stage, it presents as thick, infiltrated plaques and tumors. Lesions are often characterized by alopecic patches on the scalp, which may become secondarily infected [4,5].

Although the literature is sparse, some dermoscopic characteristics have been reported for FMF: comedo-like openings, white halos around the follicle, white structureless areas, and dotted and fine linear vessels [6,7]. In a patient with scalp alopecia due to FMF, dermoscopy showed milky-white globules and black dots/broken hairs, while in a patient with spiky MF, keratotic cone-shaped spicules were noted [8,9].

In fact, dermoscopy has been found to be helpful in assessing early MF [10]. Orange-yellow and white patches, short, fine and linear vessels, and perifollicular white scales have been described as specific dermoscopic features of early MF [10,11]. In our case, the unusual feature lies in the clinical presentation of diffuse comedo-like lesions and the appearance of a rapidly progressive alopecic plaque. Dermoscopy also allowed us to question the diagnosis of AD, given the presence of a white halo around follicles, comedo-like openings, and dotted and short linear vessels.

Lesions can mimic a variety of follicular dermatoses, including infectious and non-infectious disorders (such as acne vulgaris, lichen spinulosus, keratosis pilaris, etc.) [5,12]. Considering its large clinical spectrum, diagnosis is challenging and requires correlation between clinical and pathological findings [6]. The histopathology of MF found on biopsy can vary according to disease stage [13]. Histopathological findings consistent with FMF include a predominance of infiltration of the epithelium of the hair follicle by neoplastic T lymphocytes, infiltration of the epidermis, and CD4 predominance on immunohistochemical study [14]. Follicular mucinosis may be associated or not. Our patient's histopathological findings revealed abnormal findings for the patch stage with moderate epidermotropic and pilotropic dermal infiltrates, no visible mucin, and a concordant immunohistochemical profile (CD3, CD4, CD8, CD20, CD2, and CD5 positive and Ki67 assessed at 10%). Our case highlights the need for histopathological analysis in patients with doubtful clinical presentation, and for biopsies to be taken from multiple sites in order to obtain an accurate diagnosis. To date, there are no guidelines specifically designed for pediatric MF. Treatment is extrapolated from adult practice, showing a good response to phototherapy in the pediatric population [12,15]. Recent studies showed that narrow band ultraviolet B (NbUVB) phototherapy is as effective as psoralen and long-wave ultraviolet radiation (PUVA) phototherapy in inducing remission in children with early folliculotropic MF, regardless of the depth of the infiltrate [15]. Other treatments are also used in the early MF stages as topical steroids, systemic treatments (IFN-α, retinoids), methotrexate, and immunotherapy (mogamulizumab and brentuximab vedotin) [16].​​​​​​ In our case, the patient was unable to benefit from phototherapy sessions due to geographical issues and was put on strong topical steroids and a low dose of methotrexate, with partial improvement after four months of treatment.

Juvenile MF seems to evolve less aggressively than in adults, and the rate of progression from early to advanced stages is very low [4]. As for FMF in particular, children generally present at an earlier stage and are more likely to have a favorable prognosis, while adults have a prognosis closer to the tumor stage of the disease [17].

## Conclusions

Juvenile FMF remains a rare and misleading condition. Physicians need to be aware of this disease to enable early diagnosis and treatment, even in children with a history of AD. This case highlights the importance of maintaining a high level of suspicion in the face of chronic eczema-like lesions and highlights the essential role of the dermoscopic tool in identifying patterns that may improve the early diagnosis of MF. Larger studies with extended follow-up periods are needed to establish specific treatment guidelines for the paediatric population.
